# Utility of sirolimus coated balloons in the peripheral vasculature – a review of the current literature

**DOI:** 10.1186/s42155-022-00308-z

**Published:** 2022-06-24

**Authors:** Y. L. Linn, E. T. C. Choke, C. J. Q. Yap, R. Y. Tan, A. Patel, T. Y. Tang

**Affiliations:** 1 Department of Vascular Surgery, Singapore General Hospital, Singapore, Singapore; 2 Department of General Surgery, Sengkang General Hospital, Singapore, Singapore; 3 Department of Nephrology, Singapore General Hospital, Singapore, Singapore; 4 Department of Vascular Interventional Radiology, Singapore General Hospital, Singapore, Singapore; 5 Duke NUS Graduate Medical School, Singapore, Singapore

**Keywords:** Sirolimus coated balloon, Paclitaxel coated balloon, Chronic limb threatening ischaemia, Peripheral arterial disease, Percutaneous transluminal angioplasty

## Abstract

Sirolimus-coated balloons (SCB) have demonstrated much promise as an alternative drug eluting device to the existing paclitaxel coated balloon platforms for the treatment of peripheral arterial disease (PAD). They have been well tested pre-clinically and have demonstrated anti-restenotic effects as well as clinical safety in its use for treatment of coronary artery disease. The existing approved SCBs have thus far demonstrated good short-term patency (12-months) and did not exhibit any major adverse events or device related shortcomings in its use for treatment of PAD. There are several studies ongoing which aim to further investigate the efficacy of existing SCBs and establish a direct comparison of its outcomes compared with plain balloon angioplasty. Also, SCB utility to salvage failing arteriovenous fistulas for haemodialysis patients has also been explored. We review the current progress made in the establishment of SCB in the treatment of PAD as well as highlight ongoing studies investigating the role of SCB in various settings.

## Introduction

Peripheral arterial disease (PAD) is a chronic atherosclerotic disease affecting the arterial vasculature of the lower limbs, resulting in progressive narrowing (Kullo and Rooke 2016). It affects more than 200 million people globally (Global burden of disease study 2013 collaborators 2015) and accounts for significant healthcare costs (Conte et al. 2019). The most severe form of PAD is known as chronic limb threatening ischaemia (CLTI), and results from occlusive arterial disease leading to tissue loss, which manifests as ischaemic rest pain, non-healing ulcers or gangrene (Hirsch et al. 2006). This has been associated with high mortality and major lower extremity amputation (LEA) rates (Reinecke et al. 2015).

The primary aim of treatment of CLTI is the achievement of revascularization in a timely manner (Hirsch et al. 2006) to aid wound healing and to minimize the risk of major lower LEA, which in itself has been associated with poorer quality of life and mobility (Mayfield et al. 2001). This has traditionally been achieved via an open bypass surgery, but the development of percutaneous transluminal angioplasty (PTA) techniques resulted in a push towards an endovascular-first approach for lower limb revascularization (Aboyans et al. 2018), where lower limb angioplasties were employed as first line treatment in the restoration of straight line pulsatile arterial flow to the foot. This has been associated with improved amputation-free survival rates (AFS) compared to traditional open surgical bypass (Lin et al. 2019). However, a major drawback of performing standard PTA, otherwise known as plain old balloon angioplasty (POBA), is the quick time to restenosis and loss of luminal patency (Varetto et al. 2019). This is related to the barotrauma created during the POBA process, causing inflammation and ultimately neointimal hyperplasia (NIH) (Biondi-Zoccai et al. 2009), resulting in higher re-intervention rates, albeit with no differences in mortality outcomes (Norgren et al. 2007).

The concept of drug coated balloons (DCB) was introduced to mitigate the effects of NIH, with a typical onset of 1 month after PTA (Braga et al. 2019). This was achieved via the anti-proliferative effects of drugs such as paclitaxel, which inhibit the NIH process and delays restenosis, resulting in improved luminal patency (Caradu et al. 2019). In particular, paclitaxel coated balloons (PCB) have been demonstrated to achieve longer term patency compared to POBA with Class IIb evidence (Salisbury et al. 2016) and have been deemed first line treatment by groups such as the Society for Cardiovascular Angiography and Interventions and European Society of Cardiology for treatment of femoro-popliteal PAD (Aboyans et al. 2018; Feldman et al. 2018).

However, the findings of the landmark meta-analysis by Katsanos et al. (Katsanos et al. 2018) in 2018 called in doubt the role and safety of the use of paclitaxel devices including PCB and stents, where it was suggested that there was an increase mortality in patients with femoropopliteal disease following treatment with paclitaxel devices in the medium term. This resulted in widespread alarm among the endovascular surgery community (Tang et al. 2020a), who have by that point assumed the safety of paclitaxel devices (Salisbury et al. 2016) and considered it standard of care over POBA for the femoropopliteal region (Aboyans et al. 2018; Feldman et al. 2018). A subsequent study by Katsanos (Katsanos et al. 2020) also demonstrated worse AFS with the use of PCB compared to POBA for below the knee (BTK) disease at 12 months. While several justifications have been offered to cast doubt on the findings of the meta-analysis (Tang et al. 2020a; Soon et al. 2020), such as the lack of standardized endpoints, poor handling of outcomes, lack of long-term data and insufficient statistical power, sufficient damage has been done, with the Food and Drug Administration (FDA) in the United States eventually publishing a provisional warning on the use of paclitaxel devices pending further recommendation (Update 2019).

## Use of Sirolimus coated balloons for peripheral arterial disease

Concurrent to the woes facing paclitaxel devices, drug coated balloons using sirolimus have also been introduced as a possible alternative to PCB. Similar to paclitaxel, sirolimus is an antiproliferative agent. It acts by reversibly placing the cell into resting phase, G_0_, retaining its viability, in contrast with the mechanism of action of paclitaxel which interferes with microtubule formation during cell division which induces apoptosis (Sehgal 2003). In contrast to paclitaxel, sirolimus also has anti-inflammatory and anti-restenotic effects, as well as a broader therapeutic range and a 100-fold higher margin of safety (Ali et al. 2019).

Sirolimus devices have thus far already been used in the treatment of coronary arterial disease (CAD), with demonstrable lower restenosis rates compared to paclitaxel devices (Abizaid 2007). The use of sirolimus eluting stents also been demonstrated to inhibit the volume of NIH at 6 months compared to bare metal stents in the treatment of CAD, with resulting reduced restenosis rates (Abizaid et al. 2004). Its efficacy was initially limited by a slower spread within the arterial wall, reducing its retention levels and resulting in rapid dilution and sub-therapeutic treatment, especially when treating the larger peripheral lower limb arteries (Lemos et al. 2013).

This was a problem especially for its use in the peripheral circulation, where ‘nude’ sirolimus application had slow tissue absorption, necessitating the use of a co-solvent to enhance tissue uptake (Tang et al. 2020a). Initial attempts also saw rapid deactivation of sirolimus molecules when delivered into aqueous media via sirolimus eluting stents in the superficial femoral artery (SFA), resulting in no or marginal benefit (Tang et al. 2020a). However, the development of novel sirolimus-delivery technologies has resulted in more effective use of sirolimus coated balloons (SCB) in the periphery, including in those patients with below the knee (BTK) tibial arterial disease (Soon et al. 2020). At present, at least 2 SCBs have been granted breakthrough device designation by the FDA for treatment of PAD – *Selution SLR™* (MA ed. Alliance, SA, Mont-sur-Rolle, Switzerland) and *MagicTouch™* (Concept Medical Inc., Surat, India) (Tang et al. 2020a).

The *Selution SLR™* combines the use of amphipathic lipid cell adherence coating with sirolimus biodegradable micro-reservoirs to increase drug uptake into the arterial wall, thereby minimizing drug loss to the circulation and achieve extended elution kinetics (Med Alliance’s 2018). The *MagicTouch™* employs the use of phospholipid to achieve 100% sirolimus sub-micron particle coating on its balloon surfaces, allowing for controlled drug delivery into the arterial wall (Lemos et al. 2013). These technologies enable maximal prolonged drug elution at therapeutic levels to minimize NIH and restenosis.

## Present evidence for use of Sirolimus devices

Extensive preclinical testing has been performed on the *Selution SLR™* SCB prior to its usage in humans, including assessment of its dimensional and functional attributes, drug and coating characterization, biological evaluation, pharmacokinetics and histological safety, sterilization, packaging integrity as well as stability (Böhme et al. 2021). In vivo animal studies were conducted in rabbit iliac arteries to assess pharmacokinetics and histological safety of up to 6 months and found the device to perform as intended with no complication such as distal emboli or infarct involving the micro-reservoirs of sirolimus used (Böhme et al. 2021). The approximate half-life of sirolimus was approximately 90 days in comparison with days to weeks for paclitaxel (Katsanos et al. 2018), conferring it the theoretical benefit of longer therapeutic effect.

Subsequently, the first-in-human trial was performed with the use of *Selution SLR™* SCB for treatment of femoropopliteal lesion, entitled “Prospective, Controlled, Multi-centre, Open, Single-Arm Clinical Investigation of the Treatment of Patients with Femoropopliteal Artery Lesions with a Novel Drug-coated Balloon” (NCT02941224) (Zeller et al. 2020) (Table 1). Fifty patients with complex SFA and popliteal artery lesions were treated with the *Selution SLR™* SCB, with a mean lesion length of 64.3 mm and 30% total complete occlusion. 6-months data found a target lesion revascularization (TLR) rate of 2.3%, no major LEA or death and improvement in 1 or more categories on the Rutherford Classification in 73% of patients. Primary patency rate as assessed by Duplex ultrasound was 88.4% and freedom from restenosis was 91.2%. 12- and 24- month data (Böhme et al. 2021) also demonstrated sustained improvement in 1 or more categories on the Rutherford Classification from baseline in 78% and 84% of patients at 12- and 24-months respectively and 85% freedom from TLR at 12-month post procedure. There were no incidence of major LEA or death at 24-months, although the patient cohort comprised of only claudicants (Böhme et al. 2021).

**Table 1:** Summary of evidence of use of sirolimus coated balloons

Study	Aims/Findings
SCB in peripheral vasculature	
NCT02941224 (Zeller et al. 2020)	Prospective, single-arm, open-label, multi-center study of 50 patients with femoropopliteal lesions treated with *Selution SLR™* demonstrating 88.4% primary patency, 91.2% freedom from restenosis and 85% freedom from TLR at 12 months.
PRESTIGE (Tang et al. 2021a)	Prospective, single-arm, multi-investigator, single-center study of 25 patients with CLTI treated with *Selution SLR™* demonstrating 100% technical success, 81.5% primary patency at 6 months and 8.3.% freedom from clinically driven TLR.
XTOSI (Choke et al. 2021)	Prospective, single-arm, open-label, single-center study of 50 patients with PAD treated with *MagicTouch™* demonstrating 100% technical and device success, 89.7% 12-month freedom from clinically driven TLR, 81.6% AFS, 92.9% limb salvage and 84.6% wound healing rate.
SCB in AVF	
ISABELLA (Tang et al. 2021b; Tang et al. 2022a)	Prospective, single-center study of 40 failing AVF treated with *Selution SLR™* (Med Alliance), demonstrating 95.1% and 71.8% primary patency at 3- and 6-month, 100% technical and procedural success.
MATLIDA (Tang et al. 2020b)	Prospective, single-center study of *MagicTouch™* for treatment of 33 failing AVF, demonstrating 97.9% and 82.9% primary patencies at 3 and 6 months, 100% technical and procedural success.
AVG (Tan et al. 2021a)	Prospective, single-center study of *MagicTouch™* for treatment of AVG at the graft-vein junction in 20 patients, finding 3- and 6-month primary patency rates of 76% and 65%.
Future studies	
LIFE-BTK (LIFE-BTK 2020)	Prospective, randomised controlled trial comparing *Espirit™* BTK device (Abbott, Chicago, Illinois) for treatment of infra-popliteal disease compared to standard PTA device.
MDK-1901 (MDK-1901 2022)	Prospective, randomised controlled trial comparing *Selution SLR™* for treatment of superficial femoral and popliteal artery lesions in PAD patients.
IMPRESSION (Sirolimus 2022)	Prospective, randomised controlled trial comparing *MagicTouch™* balloon with standard POBA in the treatment of failing AVF on the rate of primary patency at 6 months.
SAVE (SAVE Trial 2022)	Prospective, randomised controlled trial comparing *Selution SLR™* balloon with standard POBA in the treatment of failing AVF, assessing the primary patency at 6 months as well as freedom from serious adverse events at 30 days.
ACELEPIOS (Taneva et al. 2022)	Prospective, randomised controlled, single-center, noninferiority study comparing use of PCB (*Ranger*, Boston Scientific) with SCB (*MagicTouch™*) for treatment of femoropopliteal lesions.
SIRONA (Teichgräber et al. 2021)	Prospective, randomised controlled, single-blinded, multi-center noninferiority study comparing use of PCB (commercially available) with SCB (*MagicTouch™*) for treatment of femoropopliteal lesions.

To study the efficacy and safety of *Selution SLR™* SCB for treatment of TASC II C and D tibial occlusive lesions in patients with CLTI, the PRESTIGE trial (NCT04071782), a pilot prospective, non randomised, single-arm, multi—investigator single-center study was carried out (Tang et al. 2021a). Twenty five patients with 33 atherosclerotic lesions of TASC II C and D aetiology were enrolled (all Rutherford 5 ft wounds). Technical success was 100% with 81.5% primary tibial patency at 6 months and 83.3% freedom from clinically driven TLR. One major LEA and 3 deaths were recorded. 81,8% of patients showed improvement by at least 1 Rutherford category by 6 months. There was a significant improvement in the EQ. 5D quality of life scores at 6 months compared to baseline and between 3 and 6 months, which may be related to wound healing and regaining independence to ambulate again. The authors concluded that *Selution SLR™* SCB was safe and efficacious in the treatment of tibial occlusive lesions with good technical and clinical success with high patency and AFS.

Preclinical animal studies on delivery of sirolimus using *MagicTouch™* have also demonstrated successful delivery of the drug into the inner layer of arterial vessels, with some degree of penetration into the adventitia (Lemos et al. 2013). Outcomes of the *MagicTouch™* SCB in the cardiac literature as captured by the Nanolute Registry also demonstrated high procedural success rates of up to 99.7% with low device-related adverse events at 4.2% and TLR at 3.6% (Dani et al. 2019). In the first direct comparison between PCB and SCB for treatment of in-stent restenosis for coronary disease, Ali et al. (Ali et al. 2019) found that sirolimus was non-inferior to paclitaxel with both DCB demonstrating equivalent 6-month performance.

12-month data from the first-in-man study of the use of *MagicTouch™* SCB in treatment of PAD including femoropopliteal and BTK disease from the XTOSI study was published in 2021 (Choke et al. 2021). This was a prospective, single-arm, single-center study, which studied 50 patients, 20 of whom had femoropopliteal disease and 30 had BTK disease. 100% technical and device success was encountered, and 12-month freedom from clinically driven TLR was 89.7%, AFS was 81.6%, limb salvage was 92.9% and 84.6% wound healing rate. No distal embolisation was reported. The authors concluded that *MagicTouch™* was safe with no early concerns and had promising primary patency.

The promising data for sirolimus devices has also led to it being considered for use in treatment of arterio-venous fistulas (AVF) and grafts (AVG). A pilot prospective single center clinical study on the use of *Selution SLR™* for treatment of failing AVF in 40 Asian patients found 95.1% and 71.8% primary patency 3- and 6-months post fistuloplasty with *Selution SLR™* with 100% technical and procedural success (Tang et al. 2021b). However, recent published data (Tang et al. 2022a) demonstrated a drop in primary patency at 12-months to 44.4%, suggesting a possible need for further drug elution into the arterial wall to inhibit NIH between the 6- and 12-month interval timepoint. A separate pilot single center study from the same group of 33 Asian patients on the use of *MagicTouch™* SCB for the treatment of failing AVF found 97.9% and 82.9% primary patencies at 3- and 6-months with 100% technical and procedural success (Tang et al. 2020b). While a follow-up study found primary patency post treatment with *MagicTouch™* SCB at 12-month to drop to 58%, this was comparable to existing data of paclitaxel devices (Tang et al. 2021c). The use of SCB for treatment of graft-vein junction post AVG thrombectomy has also been studied, with a single center prospective pilot study of 20 patients finding 3- and 6-month primary patency rates post treatment with *MagicTouch™* SCB at the graft-vein junction to be 76% and 65% respectively (Tan et al. 2021a).

## Discussion

### Future studies in the pipeline

The interest in the use of sirolimus products in the treatment of PAD has led to several studies being conducted to investigate its efficacy and safety. The LIFE-BTK trial (LIFE-BTK 2020) (NCT04227899) is an ongoing randomized control trial comparing the use of the Espirit™ BTK device (Abbott, Chicago, Illinois) for treatment of infra-popliteal disease compared to standard PTA device. Its estimated enrollment is 225 participants and aimed to study the 6-month limb salvage and primary patency as well as freedom from major adverse events at 6 months and peri-operative death at 30 days. The Japanese MDK-1901 clinical study (MDK-1901 2022) (JapicCTI-205,434) is another ongoing trial assessing the efficacy of Selution™ for treatment of superficial femoral and popliteal artery lesions in a Japanese PAD population for device registration in Japan. The primary endpoint was the primary patency rate of the target lesion at 12-month, and the secondary endpoint was the efficacy and safety as assessed via the technical, procedural and clinical success, TLR, major adverse events and death.

Several ongoing studies also exist, which aim to study the use of sirolimus balloons for treatment of failing AVF. The IMPRESSION trial (Sirolimus 2022) (NCT04409912) aims to compare the use of *MagicTouch™* balloon with standard POBA in the treatment of failing AVF on the rate of primary patency at 6 months. The ongoing SAVE trial (SAVE Trial 2022) (NCT04327609) attempts to compare the use of *Selution SLR™* balloon with standard POBA in the treatment of failing AVF, assessing the primary patency at 6 months as well as freedom from serious adverse events at 30 days. This is a joint collaboration between Greece and Singapore and will enable to look at potential ethnic differences in AVF salvage presentation and outcomes.

### Slow flow phenomenon with SCBs?

One issue pertaining to the use of DCB is the potential for the slow-flow phenomenon (Tang et al. 2021d). This was thought to be due to particulate embolization with the application of DCB with over 50% of drug lost downstream reported (Torii et al. 2018). This was previously established to have affected the use of PCB, with up to 8% incidence with its use for the treatment of PAD with associated reduction in freedom from TLR, AFS and overall survival (Tang et al. 2021d). This was also thought to be a possible cause for the poorer outcomes with paclitaxel devices use reported in the meta-analyses by Katsanos et al. (Katsanos et al. 2018; Katsanos et al. 2020). Early studies suggest no evidence of slow-flow phenomenon with the use of SCB in the treatment of PAD, even for treatment of below ankle disease, as evidenced by review of angiographic images post treatment with drug elution (SAVE Trial 2022). This was attributable to the micro-reservoirs of phospholipid polymer complex with the cell adherent technology of the *Selution SLR™* SCB, which minimizes distal embolization.

### Regulatory issues

Paclitaxel coated DCB for treating peripheral atherosclerotic disease were initially approved in Europe in 2012 and approved for use by FDA in 2014. DCBs quickly became the standard of care for treating the SFA in Europe, Asia and USA, and other indications including coronary in stent restenosis and infrapopliteal diseases are currently undergoing regulatory trials and are expected to be approved for marketing in the near future, While currently approved devices in USA all use paclitaxel as the anti-restenotic agent great progress has been made in developing the “Limus” class of antirestenotic agents which are welcomed by the regulatory bodies for improved performance and known safety characteristics. New DCBs on the approval pathway mainly feature this new class of antirestenotic issues. At present, only the *MagicTouch™* and the *Selution SLR™* DCB have received approval for commercial distribution in Europe and granted FDA approval as a breakthrough device for treatment of lower limb PAD (Böhme et al. 2021). Reimbursement for its use have thus far been variable.

### Paclitaxel or Sirolimus

The role of sirolimus DCBs in treatment of disease in the peripheral vasculature is promising, although more data is required to establish its long-term efficacy and safety. Whether sirolimus DCBs will eventually replace paclitaxel DCBs in treatment of the peripheral vasculature will depend on future findings for both DCBs. Sirolimus has the theoretical advantage over paclitaxel for its anti-inflammatory and anti-restenotic effects, as well as a broader therapeutic range (Ali et al. 2019). At present, the ACELEPIOS study (Taneva et al. 2022), a prospective, randomized controlled single center non inferiority study, which attempted to compare procedural success and primary patency as well as 12 month-freedom from MAE, procedural success, and improvement in Rutherford category post treatment with paclitaxel DCB (Ranger, Boston Scientific) compared to sirolimus DCB (*MagicTouch™*) for femoropopliteal lesions, released preliminary data of six patients (three treated with PCB and three with SCB) demonstrated safety and efficacy of both PCB and SCB. This is at present the first-in-literature data and its eventual findings would be helpful in shaping the role of sirolimus DCB in the peripheral vasculature. Another study, the SIRONA trial (Teichgräber et al. 2021), a single-blind, multi-centre, randomized controlled noninferiority study, which aims to investigate the safety and efficacy of range of commercially available paclitaxel DCB compared to sirolimus DCB (*MagicTouch™*) in treatment of the femoropopliteal artery, is also presently in recruitment phase. These studies would be a first step in determining the place of sirolimus DCB among its contemporaries. An IDE BTK RCT comparing Selution™ and POBA is about to start with lead centres in the US, Europe and Asia. All these studies will need to take note the lessons of the paclitaxel-based studies in PAD where follow-up was at best variable and patient level follow-up data missing. More pre-clinical testing of the SCBs in different animal models are also required especially for the AVF and PAD settings to look at pharmarcokinetics of the balloon. More data looking at the use of SCBs in different parts of the AVF circuit are warranted especially as we are beginning to realise that drug may have differential effects on different types of stenotic lesions (Tan et al. 2021b).

In terms of dosage, Selution™ uses 1mcg/mm^2^ and *MagicTouch™* uses 1.25mcg/mm^2^. These doses are both lower than used in paclitaxel DCB, although the technology to introduce molecular sirolimus into the arterial wall differs between sirolimus and paclitaxel DCB. Hence, calcium, which may be an issue for paclitaxel DCB (Fanelli et al. 2014), may not be of concern for sirolimus DCB since sirolimus performs with reversible binding unlike paclitaxel and may allow better penetration into the arterial wall. Will this will have to be proven, present outcome data from PRESTIGE and XTOSI are encouraging especially for BTK vessels (Tang et al. 2021a; Choke et al. 2021). In terms of treating long lesions, treatment with stents may not be suitable as long stents have issues such as occlusion, in particular in Asian vessels which are smaller than Caucasian vessels, and can be very difficult to reopen when occluded (Soon et al. 2021). While there is a potential for distal embolisation and outflow obstruction resulting in impaired wound healing and increased LEA as with paclitaxel DCB, there have thus far been no slow flow phenomenon with sirolimus SCB (Tang et al. 2022b).

There are ultimately still many challenges in the quest to improve revascularization outcomes, especially in the BTK region. The unet need is to find durable revascularization without restenosis, managing dissection as well as recoil and crossing and treating heavily calcified lesions. Furthermore, we need better foor perfusion imaging techniques pre and post intervention to guide us on targeted wound debridement to achieve optimal wound healing outcomes in the setting of CLTI. There have been a huge interest in the role of drug coated technology to minimize the risk of restenosis and therefore reduce potential reintervention but DCB really only addresses the NIH aspect and not the recoil and dissection, which require saffolds and tacks to address the mechanical problem. In the next 2 to 5 years, the answer using sirolimus will become obvious with all the trials coming to fruition and having learnt from the paclitaxel issue. Prospective follow up of patients to give patient level data will be of importance. It is unlikely that sirolimus technology can answer all the problems and that a toolbox of multiple technologies including stents (for scaffolding), atherectomy to address the calcium issue and DCB to minimize NIH will be required.

## Conclusion

We conclude that sirolimus DCBs represent a promising alternative in treatment of disease of the peripheral vasculature, although further studies are required to establish its clinical efficacy and safety as well as cost effectiveness in terms of mortality benefits and reduced time to reintervention compared to PCB and plain POBA.

## Data Availability

All data generated or analysed during this study are included in this published article.

## Abbreviations

SCB: sirolimus-coated balloon
PAD: peripheal arterial disease
CLTI: chronic limb threatening ischaemia
LEA: lower extremity amputation
PTA: percutaneous transluminal angioplasty
AFS: amputation free survival
POBA: plain old balloon angioplasty
NIH: neointimal hyperplasia
DCB: drug coated balloon
PCB: paclitaxel coated balloon
BTK: below the knee
FDA: food and drug administration
CAD: coronary arterial disease
SFA: superficial femoral artery
TLR: target lesion revascularisation
AVF: arterio-venous fistula
AVG: arterio-venous graft
